# Sex Differences in the Peripheral Immune System in Patients with Depression

**DOI:** 10.3389/fpsyt.2017.00108

**Published:** 2017-06-16

**Authors:** Badari Birur, Ellen M. Amrock, Richard C. Shelton, Li Li

**Affiliations:** ^1^Department of Psychiatry and Behavioral Neurobiology, University of Alabama at Birmingham, Birmingham, AL, United States

**Keywords:** sex, depression, depressive symptoms, inflammation, inflammatory markers

## Abstract

**Background:**

Females are twice as likely as males to experience depression. Recent findings indicate a relationship linking inflammation with depression. Whether the higher prevalence of depression in women is sex-specific or if inflammation contributes to a higher prevalence of depression in females is unclear. Thus, the objective was to determine whether depressed females show higher inflammation compared to males in a cross-sectional study.

**Materials and methods:**

Two hundred participants were enrolled. Depressive symptoms were assessed using the Montgomery–Åsberg Depression Rating Scale (MADRS), and blood samples were collected from all participants to measure inflammatory blood markers.

**Results:**

Higher rates of suicidal thoughts, pessimism, and lassitude measured by the MADRS were seen in depressed females compared with depressed males. Among all inflammatory markers measured, there were no significant differences in depressed males vs. male controls. Increased levels of interleukin (IL)-8, interferon-γ, and leptin, and decreased levels of IL-5 and adiponectin were observed in depressed females compared to female controls. Compared with depressed males, IL-6 and leptin levels were significantly elevated in depressed females after controlling for body mass index. Correlation analysis revealed that depression severity negatively correlated with IL-12 in males, and positively correlated with IL-1β and tumor necrosis factor (TNF)-α in females. IL-1β and TNF-α correlated with suicidal thoughts, lassitude, and pessimism in depressed females.

**Conclusion:**

Our findings indicate a sex-specific relationship between inflammation and depression, which may be important in identifying potential psychopathology and suggesting novel immunomodulatory treatments for depressed females.

## Introduction

Systemic inflammation contributes to the development of major depressive disorder (MDD) and suicidality in some individuals. Subjects with inflammatory diseases are three to four times more likely to experience MDD (1). Patients with major depression also often show increased inflammatory markers, including C-reactive protein (CRP), interleukin-6 (IL-6), and tumor necrosis factor (TNF)-α, relative to controls (2–5). Thus, inflammation appears to be an established mechanism in the pathogenesis of MDD (6, 7). Despite the fact that inflammation seems to be related to depression, the exact mechanism underlying this is not completely understood, and more research is needed to establish this relationship clearly.

Females are twice as likely as males to experience MDD (8–10). Interestingly, women also demonstrate a higher prevalence of autoimmune disorders when compared to men (11, 12). However, previous studies examining the role of sex in the relationships between inflammatory markers and depression have yielded inconsistent results. Some studies found higher CRP levels in depressed men than depressed women (3, 13–15), while another showed the opposite (16). In a sample of older adults with depression, researchers found that the serum levels of IL-6 were higher among men, but not women (17). Alternatively, some studies showed no difference in inflammatory markers between depressed men and women (2, 18). Therefore, possible sex differences in inflammation between males and females with MDD remain unclear.

Suicidal ideation or behavior is commonly associated with MDD. Suicide is the 10th leading cause of death in the United States, and the rate has been growing over the last 20 years (19). Thus, identifying risk factors for suicide in depressed patients is an area of increasing public health concern. There is limited evidence suggesting that some inflammatory markers such as IL-2, IL-6, IL-8, and TNF-α may play a role in the pathophysiology of suicidal behavior (20). Very few studies have examined inflammatory changes associated with suicidal behavior (21). An initial study reported elevated concentrations of soluble IL-2 receptors in suicide attempters (22) and another study reported elevated IL-6 in the cerebrospinal fluid of suicidal patients (23). However, studies examining the relationship between inflammatory markers and suicide in depressed patients in a sex-specific manner are seldom reported. In depressed patients with suicidal ideation, lassitude and pessimism are prominent, and also contribute to suicidal desires and attempts. Very little attention is focused on whether inflammatory markers are related to lassitude and pessimism in patients with depression (24).

Since females are more susceptible to MDD (25) and even healthy women show enhanced susceptibility to inflammatory disease (26), it is important to examine differences in inflammatory markers between depressed women and men. Recent inconsistent findings in the relationships between inflammation and depression also highlight the need to further explore the role of sex in their relationship. Furthermore, identification of inflammatory markers for depression and suicidal behavior in a sex-specific manner could provide a better understanding of pathophysiological processes involved, thus expanding opportunities for treatment. The primary objective of this study was to determine whether females show higher inflammation when compared to males in MDD. A secondary aim was to evaluate the relationship between individual depressive symptoms measured by the Montgomery–Åsberg Depression Rating Scale (MADRS) and inflammatory markers in females and males.

## Materials and Methods

### Subject Characteristics

Participants were recruited from the outpatient and inpatient settings at the University of Alabama at Birmingham (UAB) as well as from the local Birmingham, AL, community. The protocol was approved by the UAB Institutional Review Board, and written informed consent was obtained from all participants. Among 234 consented participants, 34 dropped due to failure to show up for a blood collection, consent withdrawal or loss to follow-up. Finally, 200 participants completed the study for data analysis. Participants were males and females aged between 19 and 65 years who were physically healthy or had stable medical conditions. Race, education level, smoking status, and age were determined by self-report. Anthropometrics were collected using National Health and Nutrition Examination Survey methods (27). Body mass index (BMI) was calculated in kilogram per square meter from height and weight measures. Subjects were diagnosed with MDD according to the Diagnostic and Statistical Manual of Mental Disorders, fourth edition (28), as confirmed by the MINI International Diagnostic Interview (29). The severity of depression was assessed using the MADRS (30). MADRS is a 10-item diagnostic questionnaire used by clinicians to assess the severity of depressive episodes in patients with mood disorders. Higher MADRS score indicates more severe depression. Each item yields a score of 0–6, and the overall score ranges from 0 to 60. The questionnaire assesses for the following symptoms: apparent sadness, reported sadness, inner tension, reduced sleep/appetite, concentration difficulties, lassitude, inability to feel, pessimistic thoughts, and suicidal thoughts. The cutoff points for total score are: 0–6 (normal/symptoms absent), 7–19 (mild depression), 20–34 (moderate depression), and >34 (severe depression) (31).

Participants were excluded if they: (1) were taking corticosteroids, antibiotics, or anti-inflammatory medications; (2) had current infectious diseases, or a history of autoimmune, endocrine or inflammatory disorders; (3) were pregnant or lactating; or (4) had a history of psychosis, bipolar disorders, illicit drugs, or alcohol abuse. They were divided into four groups: males with MDD, females with MDD, male control group, and female control group as shown in Table 1.

**Table 1:** Demographic data of participants.

	CTL male	MDD male	*P*_1_ values	CTL female	MDD female	*P*_2_ values	*P*_3_ values
Number	30	42	n/a	67	61	n/a	n/a
Age	35.2 ± 2.6	43.9 ± 1.9	0.01	37.1 ± 1.4	43.8 ± 1.6	0.03	0.30
Race (C/AA)	18/12	23/19	0.17	31/39	29/32	0.61	0.43
BMI	28.9 ± 1.1	26.7 ± 0.8	0.35	30.4 ± 1.1	31.1 ± 0.9	0.67	0.02
Education, % (≥10 years)	63%	62%	1.00	84%	69%	0.06	0.53
Smoker (yes/no)	10/20	18/24	0.47	13/54	18/43	0.22	0.21

*CTL, control; MDD, major depressive disorder; C, Caucasian; AA, African-American; BMI, body mass index; P_1_, depressed males vs. male controls; P_2_, depressed females vs. female controls; P_3_, depressed females vs. depressed males*.

### Serum Measures

Ten milliliters of blood were drawn from each participant and centrifuged at 3,000 *g* for 10 min, immediately divided into aliquots, and frozen at −80°C until analysis. The analysis of the inflammatory markers, including interferon-γ (IFN-γ), IL-1β, IL-2, IL-5, IL-6, IL-8, IL-10, IL-12 p70, IL-17, and TNF-α, was performed from blood samples using Meso Scale Discovery multiplex assay (Gaithersburg, MD, USA). Their levels were expressed in pg/ml. CRP was analyzed using immunoassay on a Stanbio Sirrus Analyzer (Stanbio Laboratory, Boeme, TX, USA) using a Pointe Scientific (Canton, MI, USA) turbidometric reagent, and was expressed in mg/L. Plasma concentrations of leptin and adiponectin were also assayed (expressed in ng/ml and μg/ml, respectively) using commercially available radioimmunoassay kits according to the procedures supplied by the manufacturer (Millipore Corp., Billerica, MA, USA). All samples were assayed in duplicate, and the mean of the duplicate values was reported.

### Statistical Analysis

All data are presented as mean ± SE unless otherwise stated. All statistical analyses were performed using the Statistical Package for the Social Sciences version 23 (SPSS Inc., IL, USA) with significance defined as *p* < 0.05. All variables were tested for normality of distribution by means of Kolmogorov–Smirnoff tests, and non-parametric tests were applied for data that were not from a normal distribution, including CRP, IFNγ, IL-1β, IL-2, IL-5, IL-6, IL-10, and IL-12. One-way analysis of variance was used to compare the age and BMI between groups. Analysis of covariance (ANCOVA) was used to compare variables of interest between depressed females/males, and the same sex controls after adjusting for age. Comparisons between depressed females and depressed males were adjusted for age and BMI, and ANCOVA was used. A chi-square test was used for categorical data. The associations between variables were tested by the Pearson correlation analysis. Missing data were handled by pairwise deletion.

## Results

### Subject Characteristics

The subject characteristics are summarized in Table 1. As shown in Table 1, depressed males and depressed females were older than their control groups. However, BMI, race, education level, and smoking status were not different between depressed patients and their controls. Depressed females and depressed males were matched in terms of age, race, education level, and smoking status, but not BMI.

### Comparison of Depressive Symptoms

**Figure 1:**
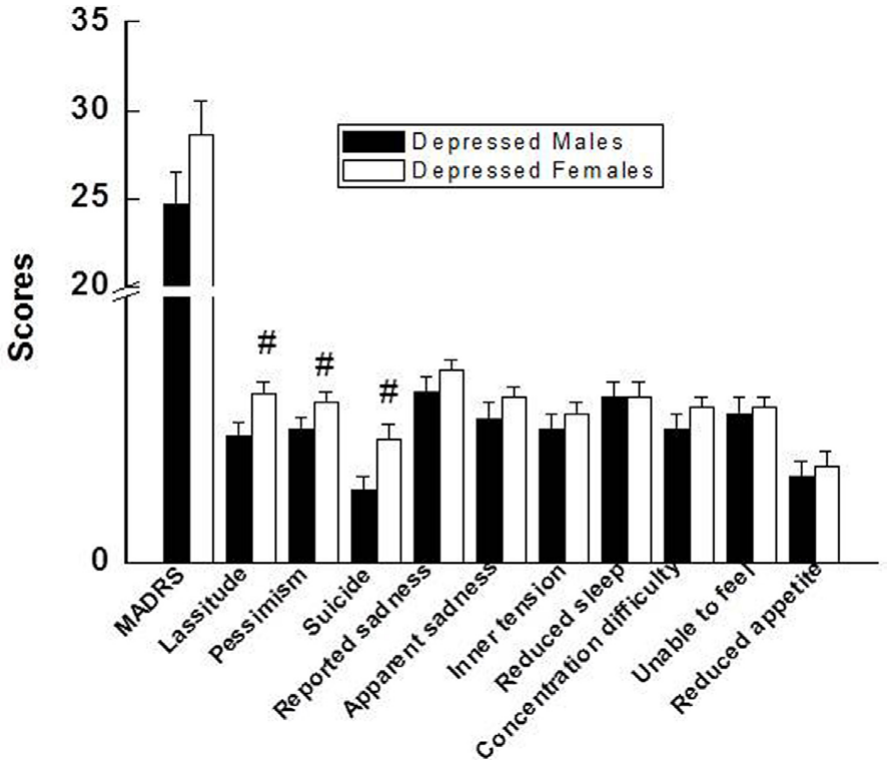
Comparison of total MADRS score and individual 10 items of the MADRS between depressed males and depressed females. Compared with depressed males, ^#^*p* < 0.05 (*p* values for lassitude, pessimism, and suicide were 0.02, 0.04, and 0.01, respectively). MADRS, Montgomery–Åsberg Depression Rating Scale.

Total MADRS scores were not different between depressed males and depressed females (Figure 1). However, among the individual items on the 10-item scale of the MADRS, depressed females scored significantly higher on the lassitude, pessimism, and suicidal thought items after controlling for BMI (Figure 1).

### Effects of Sex on the Inflammatory Factors in Patients with MDD

**Figure 2:**
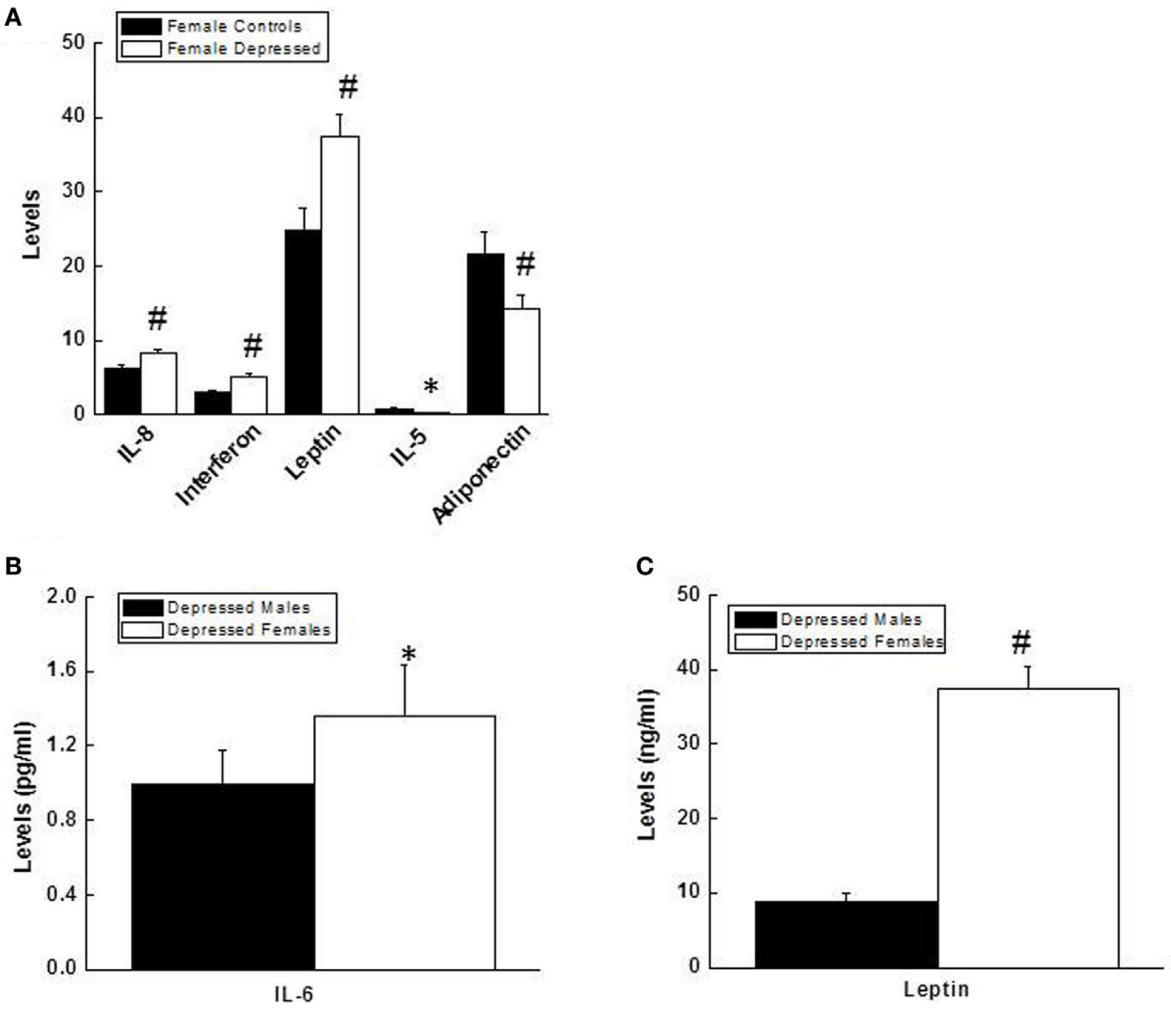
**(A)** Comparison of levels (pg/ml) for IL-8 (*p* = 0.001), interferon γ (*p* = 0.005), leptin (*p* = 0.003), IL-5 (*p* = 0.02), and adiponectin (*p* = 0.003) between depressed females and female controls. **(B,C)** Comparison of levels for IL-6 (pg/ml, *p* = 0.012), **(B)** and leptin (ng/ml, *p* = 0.000), **(C)** between depressed males and depressed females. Data are presented as mean ± SE. Compared with female control group in **(A)**, or with depressed males in **(B,C)**, *p < 0.05; ^#^*p* < 0.01. IL, interleukin.

There were no significant differences between depressed males and control males on any inflammatory marker that were measured. In contrast, depressed females showed higher levels of IL-8, IFN-γ, and leptin, and lower levels of IL-5 and adiponectin than control females (Figure 2). A similar comparison between depressed males vs. depressed females revealed significantly higher levels of IL-6 and leptin in females, which remained significant after controlling for BMI (Figure 2).

### Relationships between the Inflammatory Factors and Depressive Symptoms

**Figure 3:**
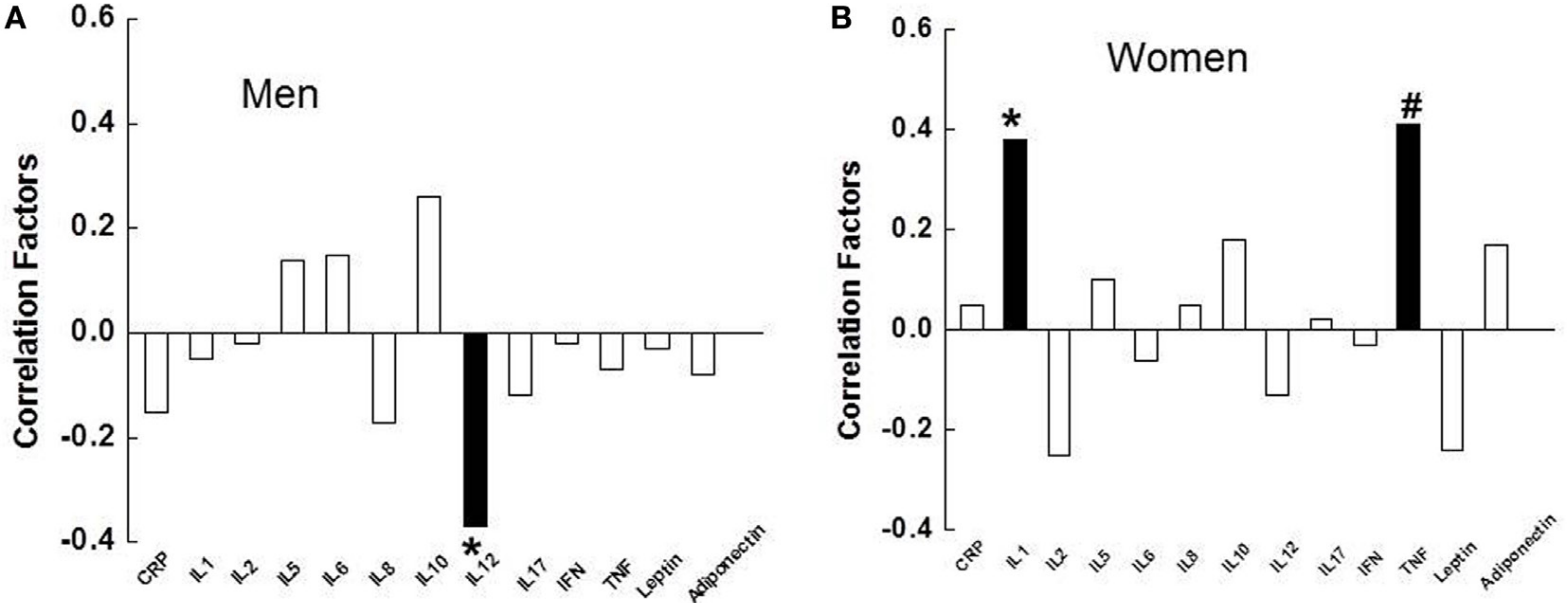
Relationships between the severity of depression, measured by the Montgomery–Åsberg Depression Rating Scale, and the inflammatory markers in depressed males **(A)**, and depressed females **(B)**. ■, significant correlation; □, non-significant. IL, interleukin; CRP, C-reactive protein; IFN, interferon; TNF, tumor necrosis factor.

Interleukin-12 negatively correlated with total MADRS score in depressed males (*p* = 0.034, Figure 3A), while IL-1β and TNF-α positively correlated with total MADRS score in depressed females (*p* = 0.012, 0.004, respectively, Figure 3B). In depressed females, but not depressed males, IL-1β and TNF-α correlated significantly with three items of the MADRS scale, including lassitude, pessimism, and suicidal thoughts (Table 2). In addition, IL-1β and TNF-α also correlated with other items of the MADRS scale, although some of the correlations were not significant as presented in Table 2.

**Table 2:** Correlational analysis between depressive symptoms and cytokines in depressed females.

	IL-1β	TNF-α
	
	*r*/*p* values	*r*/*p* values
Lassitude	0.35/0.02*	0.34/0.02*
Pessimism	0.33/0.03*	0.34/0.02*
Suicide	0.26/0.06	0.33/0.03*
Reported sadness	0.34/0.02*	0.41/0.004^#^
Apparent sadness	0.30/0.05	0.44/0.002^#^
Inner tension	0.33/0.03*	0.21/0.16
Reduced sleep	0.21/0.18	0.28/0.05
Concentration difficulty	0.35/0.02*	0.18/0.24
Unable to feel	0.28/0.07	0.24/0.11
Reduced appetite	0.30/0.05	0.36/0.01*

*IL, interleukin; TNF, tumor necrosis factor*.

*p < 0.05.

*^#^p < 0.01*.

## Discussion

The primary objective of this study was to determine if females show a greater increase in the inflammatory markers compared to males in MDD. We found that certain inflammatory markers, including IL-8, IFNγ, and leptin, were significantly elevated in depressed females compared to female controls, and IL-5 and adiponectin lower in depressed females. However, no significant differences were observed in any measured inflammatory markers between depressed males and male controls. Depressed females had higher levels of IL-6 and leptin compared to depressed males after controlling for BMI. In depressed females, but not depressed males, levels of IL-1β and TNF-α positively correlated with the severity of overall depression measured by the MADRS, and also with three individual items of the MADRS: lassitude, pessimism, and suicidal thoughts. Our study supports the notion that inflammation is related to depression; however, the association is possibly sex-specific.

Previous studies have demonstrated that MDD patients have elevated inflammatory factors including CRP, IL-6, and TNF-α compared to healthy controls (4, 5, 32). Elevated inflammation might contribute to the onset of depression in some individuals. For example, a study showed that healthy women with higher CRP exhibited more depressive symptoms over a 7-year follow-up period when compared to women with lower initial CRP values (33). Other longitudinal studies also showed that elevated CRP and IL-6 increase risk for depressive symptoms over time (34). A multiple linear regression analysis was run, which revealed that IL-1β could predict depression in females [*F*(1, 44) = 12.09, *p* < 0.01] with *R*^2^ of 0.27. Elevated pro-inflammatory markers in depressed women could contribute to the increased risk of depression in females. However, a more rigorous longitudinal study is warranted to elucidate this cause and effect relationship between the inflammatory markers and depression, especially in females.

Previous studies comparing inflammatory markers in depressed men and women yielded inconsistent findings. CRP and IL-6 were found to be higher in depressed males than depressed females in several studies (13, 14, 17). However, these results were not supported by another study that did not find an association between depression and inflammation in males (35). Among all of the inflammatory markers measured in our study, IL-6 and leptin levels were elevated in depressed females when compared with depressed males after controlling for BMI. One possibility of the discrepancy in the findings could be due to variables such as BMI, race, and age. Indeed one of our previous studies found that higher levels of circulating IL-6 and CRP in MDD patients may be explained, at least in part, by obesity (36). Therefore, it is critical to incorporate confounding variables such as BMI when the relationship between the inflammatory markers and depression is examined.

Depressed females demonstrated higher levels of suicidal ideation, pessimism, and lassitude on the MADRS when compared with depressed males. Interestingly, these three items of the MADRS also had a positive correlation with IL-1β and TNF-α in female patients. It is possible that inflammation may be a contributing factor for these three symptoms in depressed females. Our observations are consistent with a recent meta-analysis study in which levels of IL-1β and IL-6 were significantly increased in blood and postmortem brain samples of patients with suicidality (patients with active suicidal ideation, history of suicide attempt, or those who had completed suicide) vs. non-suicidal patients (37).

Females have a higher prevalence of depression than males, and as a result, they receive antidepressants to a greater extent. Depressed females report more atypical mood symptoms than men, mainly, hypersomnia, and hyperphagia. Females are more likely to experience somatic symptoms such as low energy, fatigue, and pain, and females have higher comorbidity with other internalizing disorders such as anxiety disorders, eating disorders, and somatic symptom disorders. Several lines of evidence suggest that females are more vulnerable to develop mood disorders following systemic inflammation (38–40). Our study found elevated inflammatory markers in depressed females when compared to depressed controls and depressed males. It is possible that elevated inflammatory markers could help predict future depression. Moreover, the identification of causal factors for elevated inflammation could lead to new therapeutic avenues in depressed females.

Several caveats need to be considered in interpreting the data in this study. The study was a cross-sectional design, and hence, a cause–effect relationship cannot be established. A longitudinal study is warranted, which could answer some of the questions in regard to the causal relationship between inflammation and MDD. The majority of the MDD participants who completed the study were taking antidepressants; however, none of the participants were taking antipsychotics, mood stabilizers, or any other medications like corticosteroids or antibiotics that are known to affect inflammation. Although there is no clear evidence or consistent reports regarding the anti-inflammatory effects of antidepressants (41), conclusive data on the relationship between inflammation and antidepressants would have been a valuable addition. Finally, other clinical variables like duration of the current depressive episode or total number of depressive episodes were not collected in our study subjects. It would be interesting to look further into how these variables could have an effect on the findings. This should be considered in future studies.

In summary, our study demonstrated that inflammation is related to depression; however, the association is sex-specific. Moreover, depressed females tend to have higher levels of pessimism, suicidal thoughts, and lassitude, which correlated with elevated cytokine levels. Understanding the influence of inflammation on women’s mental health may help advance our understanding of sex differences in depression, as well as assist in choosing effective antidepressants in the future. More research is needed to evaluate the role of sex in understanding the link between inflammation and MDD in order to conceptualize pathophysiology and to develop better treatments for depressed women and men.

## Ethics Statement

The study was carried out in accordance with the recommendations of Institutional Review Board at the University of Alabama at Birmingham, and Ethics committee with written informed consent from all subjects.

## Author Contributions

BB, EA, RS, and LL equally contributed to the preparation of this manuscript. LL contributed to the design, interpretation, and analysis of the study.

## Conflict of Interest Statement

The authors declare that the research was conducted in the absence of any commercial or financial relationships that could be construed as a potential conflict of interest.
